# Supplementary material for: Splice-Junction-Based Mapping of Alternative Isoforms in the Human Proteome
Source: Cell Rep. Author manuscript; Available in PMC 2020 Jan 15. (PMC6961840; doi:10.1016/j.celrep.2019.11.026)
Supplement: 2 [file NIHMS1546469-supplement-2.zip › DF1/PXD006675/InferiorVenaCava/InferiorVenaCava_5_KANK1_NLQFVGINGGYELSEK.pdf]

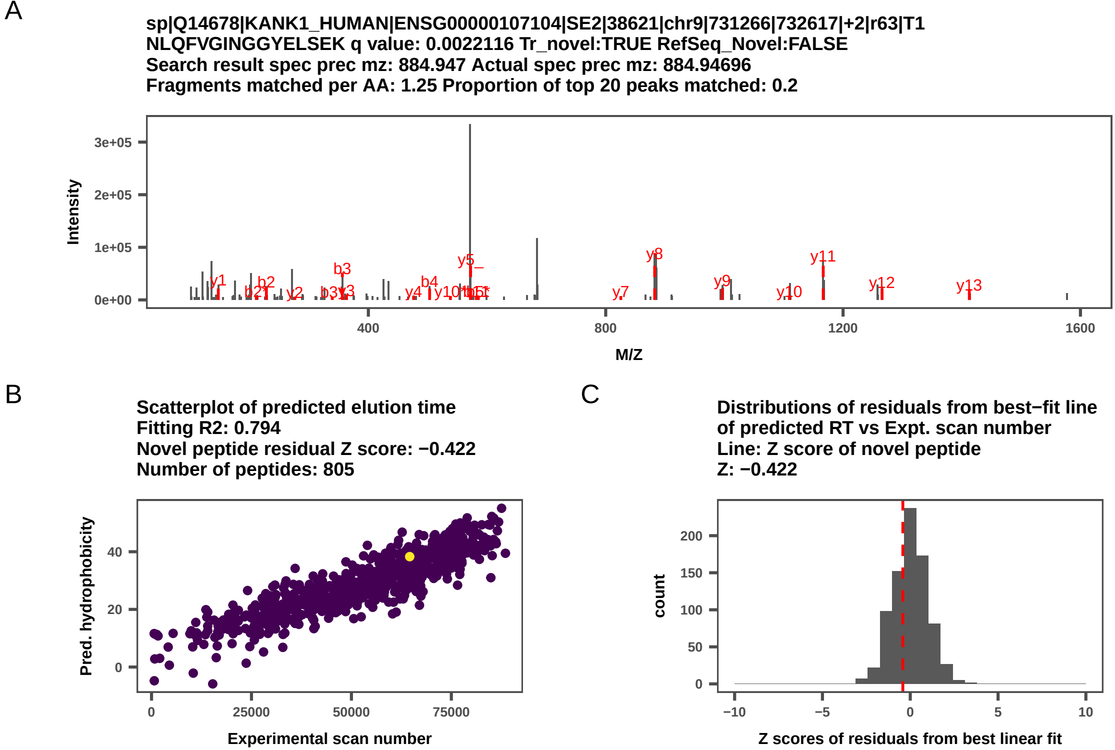
A
sp|Q14678|KANK1_HUMAN|ENSG00000107104|SE2|38621|chr9|731266|732617|+2|r63|T1
NLQFVGINGGYELSEK q value: 0.0022116 Tr_novel:TRUE RefSeq_Novel:FALSE
Search result spec prec mz: 884.947 Actual spec prec mz: 884.94696
Fragments matched per AA: 1.25 Proportion of top 20 peaks matched: 0.2
Intensity
3e+05
2e+05
1e+05
0e+00
y1
b2
b3
y2
b4
y5_
y8
y11
y9
y12
y13
y7
y10
400
800
1200
1600
M/Z
B
Scatterplot of predicted elution time
Fitting R2: 0.794
Novel peptide residual Z score: −0.422
Number of peptides: 805
Pred. hydrophobicity
40
20
0
0
25000
50000
75000
Experimental scan number
C
Distributions of residuals from best−fit line
of predicted RT vs Expt. scan number
Line: Z score of novel peptide
Z: −0.422
count
200
150
100
50
0
−10
−5
0
5
10
Z scores of residuals from best linear fit
